# Pharmacological treatment for obstructive sleep apnea: A systematic review and meta-analysis

**DOI:** 10.1016/j.clinsp.2024.100330

**Published:** 2024-02-10

**Authors:** Maria Luísa Nobre, Ayane Cristine Alves Sarmento, Priscila Farias de Oliveira, Felipe Ferreira Wanderley, José Diniz Júnior, Ana Katherine Gonçalves

**Affiliations:** a Programa de Pós-Graduação em Ciências da Saúde, Universidade Federal do Rio Grande do Norte, Natal, RN, Brazil; b Department of Clinical Analysis and Toxicology, Universidade Federal do Rio Grande do Norte, RN, Brazil; c Department of Surgery, Universidade Federal do Rio Grande do Norte, Natal, RN, Brazil; d Faculdade de Medicina, Universidade Federal do Rio Grande do Norte, Natal, RN, Brazil; e Department of Gynecology and Obstetrics, Universidade Federal do Rio Grande do Norte, Natal, RN, Brazil

**Keywords:** Obstructive Sleep Apneas, Drug therapies, Polysomnography, Systematic review

## Abstract

**Objective:**

Summarize the evidence on drug therapies for obstructive sleep apnea.

**Methods:**

The Preferred Reporting Items for Systematic Reviews and Meta-Analyses (PRISMA) guidelines were followed. PubMed, Embase, Scopus, Web of Science, SciELO, LILACS, Scopus, Cochrane Central Register of Controlled Trials, and ClinicalTrials.gov were searched on February 17th, 2023. A search strategy retrieved randomized clinical trials comparing the Apnea-Hypopnea Index (AHI) in pharmacotherapies. Studies were selected and data was extracted by two authors independently. The risk of bias was assessed using the Cochrane Risk of Bias tool. RevMan 5.4. was used for data synthesis.

**Results:**

4930 articles were obtained, 68 met inclusion criteria, and 29 studies (involving 11 drugs) were combined in a meta-analysis. Atomoxetine plus oxybutynin vs placebo in AHI mean difference of -7.71 (-10.59, -4.83) [Fixed, 95 % CI, I2 = 50 %, overall effect: *Z* = 5.25, *p* < 0.001]. Donepezil vs placebo in AHI mean difference of -8.56 (-15.78, -1.33) [Fixed, 95 % CI, I2 = 21 %, overall effect: *Z* = 2.32, *p* = 0.02]. Sodium oxybate vs placebo in AHI mean difference of -5.50 (-9.28, -1.73) [Fixed, 95 % CI, I2 = 32 %, overall effect: *Z* = 2.86, *p* = 0.004]. Trazodone vs placebo in AHI mean difference of -12.75 (-21.30, -4.19) [Fixed, 95 % CI, I2 = 0 %, overall effect: *Z* = 2.92, *p* = 0.003].

**Conclusion:**

The combination of noradrenergic and antimuscarinic drugs shows promising results. Identifying endotypes may be the key to future drug therapies for obstructive sleep apnea. Moreover, studies with longer follow-up assessing the safety and sustained effects of these treatments are needed.

**PROSPERO registration number:**

CRD42022362639.

## Highlights

• Obstructive sleep apnea affects one billion people worldwide and is associated with cardiometabolic risk and cognitive impairment.
• Drug therapy for the management of sleep apnea has been investigated, but no robust evidence that supports its benefits has been found to date.
• The combination of noradrenergic and antimuscarinic drugs shows promising results.

## Introduction

Obstructive Sleep Apnea (OSA) is a condition in which repetitive upper airway closure occurs during sleep, leading to decreased oxygen saturation and impaired sleep architecture.^1^ It is estimated to affect one billion people worldwide^2^ and is associated with cardiometabolic risk and cognitive impairment.^3^

There are many treatments for OSA, such as behavioral measures, myofascial exercises, oral appliances, surgeries, Positive Airway Pressure (PAP), and hypoglossal nerve stimulators.^4^ Although PAP treatment remains the leading choice for moderate and severe OSA, its adherence rate is low.^5^

Recent research on the pathophysiology brought light to possible targets for pharmacotherapy.^6^ The OSA pathophysiological traits (endotypes) are the anatomy of the upper airway susceptible to collapse; the poor pharynx dilator muscle responsiveness; the low arousal respiratory threshold; and the oversensitive ventilatory control system (high loop gain).^7^

Drug therapy for the management of sleep apnea has been investigated, but no robust evidence that supports its benefits has been found to date.^8^

The aim of this systematic review and meta-analysis is to summarize the evidence on pharmacotherapy for the treatment of OSA in adults.

## Methods

This review follows the Preferred Reporting Items for Systematic Reviews and Meta-Analyses (PRISMA) guidelines.^9^ The protocol is registered with the International Prospective Register of Systematic Reviews (PROSPERO CRD42022362639).

### *Inclusion criteria*

Randomized Clinical Trials (RCT) compared the Apnea-Hypopnea Index (AHI) of pharmacotherapies for adults with OSA.

### *PICOT strategy*

- Population/Participants: Adults diagnosed with OSA.
- Intervention: Any drug therapy intended to treat OSA.
- Comparator/Control: Placebo.
- Outcomes: AHI.
- Type of study: RCT.

### *Patient and public involvement*

There was no patient or public involvement.

### Search strategy

PubMed, Embase, Scopus, Web of Science, SciELO, LILACS, Scopus, Cochrane Central Register of Controlled Trials, and ClinicalTrials.gov were searched with no limitations to date or language. All electronic databases were searched on February 2023. The search strategy to be used in PubMed is presented in Table 1.

**Table 1:** Search Strategy for PubMed.

	MeSh Terms and Keywords
1	Sleep apnea syndromes
2	Obstructive sleep apnea
3	Sleep apnea
4	OR / 1‒3
5	Drug therapy
6	Pharmaceutical preparations
7	OR / 5‒6
8	Polysomnography
9	Death
10	Myocardial Infarction
11	Stroke
12	Adverse effects
13	Health-related quality of life
14	Sleep quality
15	Weight loss
16	Oximetry
17	OR / 8‒16
18	4 AND 7 AND 17

### *Data collection and analysis*

The articles were imported to Rayyan, and duplicates were removed. Two authors independently screened by title, abstract, and full text to determine inclusion criteria. A third reviewer resolved the discrepancies.

### *Data extraction and management*

Two independent authors extracted data from the included studies. The latter were inserted into a database. Meta-analysis was conducted for the studies that could be combined.

### *Risk of bias assessment*

Two reviewers independently assessed the risk of bias using the Cochrane Risk of Bias Tool (RoB 2).^10^ Each study was evaluated for the randomization process, deviations from intended interventions, missing outcome data, measurement of the outcome, and selection of the reported results.

### *Assessment of heterogeneity*

The I^2^ statistics were used to assess heterogeneity, below 25 % was considered low heterogeneity, between 25 % and 50 % moderate heterogeneity, and above 50 % high heterogeneity.

### *Measures of the treatment effect*

AHI was extracted as a continuous variable, and the mean difference with a 95 % Confidence Interval was used. This was performed using Review Manager (RevMan 5.4) software.

### *Analysis*

RevMan 5.4 was used to perform the statistical analysis. In the heterogeneity assessment, when I^2^ was > 50 %, a random-effects model was used, otherwise, a fixed-effect model was applied.

### *Grading quality of evidence*

The Grading of Recommendations Assessment Development and Evaluation (GRADE) approach was used to evaluate the strength of the evidence of the systematic review results.^11^

## Results

**Fig. 1:**
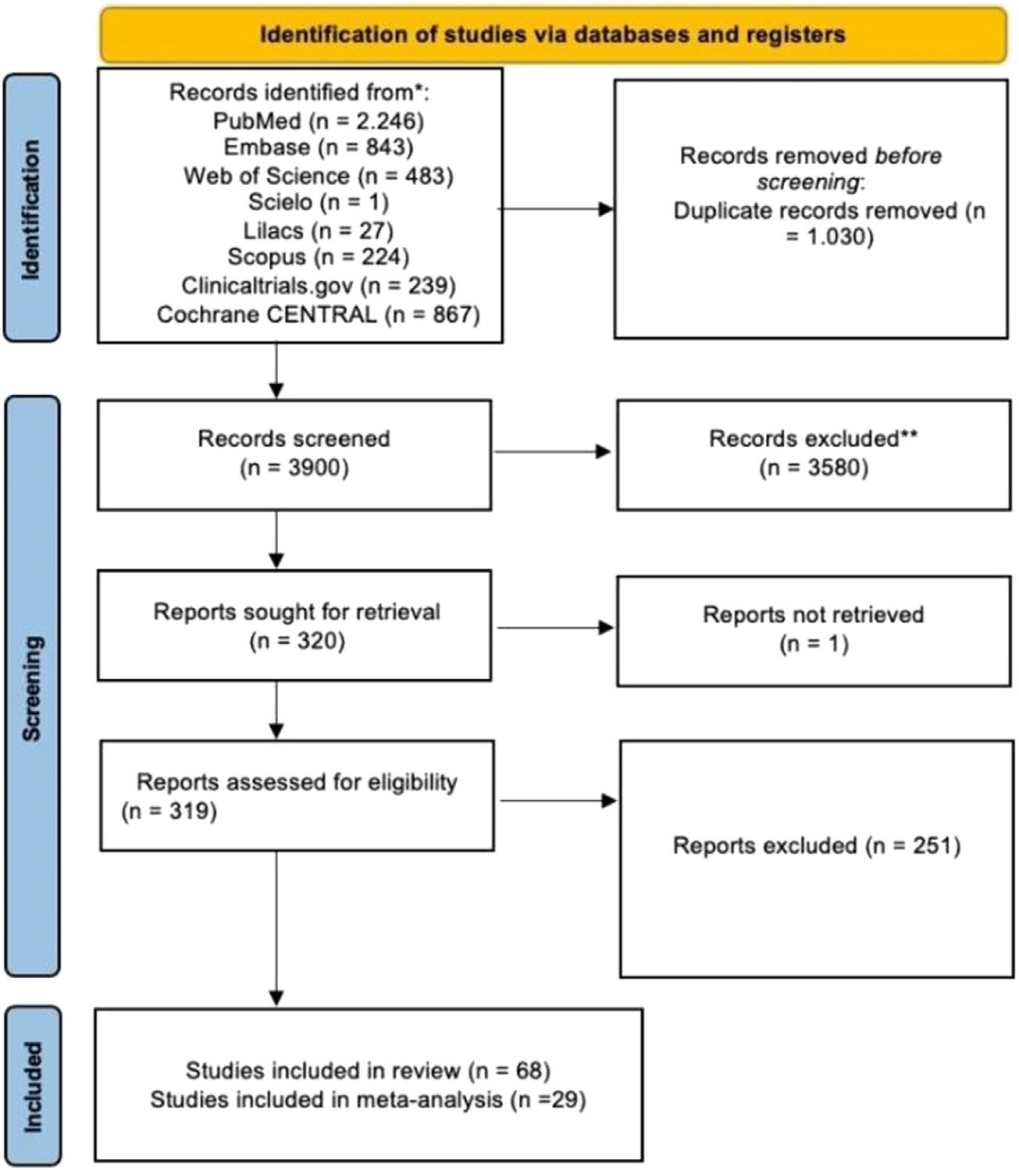
Preferred Reporting Items for Systematic Reviews and Meta-Analyses flow diagram of the selection process.

The database search retrieved 4930 articles, duplicates were removed, and two independent authors screened 3900 titles, 319 were assessed for eligibility by abstract. 68 of which met the inclusion criteria, and finally, 29 studies could be combined in the meta-analysis (11 drugs). The PRISMA flow diagram summarizes the selection process (Fig. 1). Qualitative synthesis is shown in Table 2.

**Table 2:** Qualitative synthesis of the included studies.

Author/Year	Design	Drug	Endotype	Follow-up	N	Main findings
Kinouchi 2023	RCT OL crossover	AtoOxy	MR	1 night	17	AtoOxy therapy does not reduce AHI in Japanese OSA patients.
Taranto-Motnemurro 2020	RCT DB crossover	AtoOxy	MR	1 night	17	AtoOxy markedly improved the measures of upper airway collapsibility.
Taranto-Motnemurro 2019	RCT DB crossover	AtoOxy	MR	1 night	20	AtoOxy greatly reduced OSA severity.
Rosenberg 2022	RCT DB crossover	AtoOxy	MR	1 night	30	AtoOxy had a statistically significant meaningful difference from placebo.
Schweitzer 2023	RCT DB crossover	AtoOxy	MR	1 night	60	AtoOxy improved AHI in patients with moderate pharyngeal collapsibility.
Li 2016	RCT DB crossover	Donepezil	LG	1 night	41	A single dose of donepezil did not appear to affect the severity of OSA.
Sukys-Claudino 2011	RCT DB parallel	Donepezil	LG	4 weeks	21	Donepezil treatment improved OSA index and oxygen saturation.
Moraes 2008	RCT DB parallel	Donepezil	LG	3 months	23	Donepezil improved AHI in patients with Alzheimer disease.
Lettieri 2008	RCT DB parallel	Eszopiclone	ArTh	1 night	79	The severity of OSA did not differ between eszopiclone and placebo.
Eckert 2011	RCT DB crossover	Eszopiclone	ArTh	1 night	17	Eszopiclone increased ArTh and lowered AHI in patients with OSA.
Rosenberg 2007	RCT DB crossover	Eszopiclone	ArTh	1 night	21	Mean total AHI was not significantly different in eszopiclone from placebo.
Kiely 2004	RCT DB crossover	Fluticasone	UAA	4 weeks	23	Intranasal fluticasone is of benefit to some patients with OSA and rhinitis.
Smith 2019	RCT DB parallel	Fluticasone /montelukast	UAA	3 months	26	No significant difference in AHI was found between treatment and placebo.
Acar 2013	RCT DB parallel	Mometasone	UAA	6 weeks	26	Treating allergic rhinitis has a positive effect on OSA severity.
Carley 2007	RCT DB crossover	Mirtazapine	MR	1 week	12	Mirtazapine lowered AHI but increased sedation and weight gain.
Marshall 2008a	RCT DB crossover	Mirtazapine	MR	2 weeks	36	Study discontinued prematurely due to trial failure and safety concerns.
Marshall 2008b	RCT DB parallel	Mirtazapine	MR	2 weeks	18	Mirtazapine did not improve OSA severity and induced weight gain.
Clarenbach 2008	RCT DB crossover	Xylometazoline	UAA	1 week	12	Nasal decongestion slightly reduced AHI.
An 2018	RCT DB crossover	Oxymetazoline	UAA	1 night	15	Improving nasal patency by decongestant could improve AHI.
Perger 2022	RCT DB crossover	ReboxOxy	MR	1 week	16	ReboxOxy did not improve OSA severity assessed by AHI, but lowered HB.
Berger 2023	RCT DB crossover	ReboxOxy	MR	1 week	15	ReboxOxy greatly decreased OSA severity and increased vigilance.
Altree 2023	RCT DB crossover	ReboxOxy	MR	1 night	16	Reboxetine as a single agent or combined with oxybutynin improves AHI.
George 2010	RCT DB crossover	Sodium oxybate /Zolpidem	ArTh	1 night	42	SXB does not negatively impact SDB but might increase central apneas.
George 2011	RCT DB parallel	Sodium oxybate	ArTh	2 weeks	48	It is not clear how sodium oxybate might reduce AHI in OSA patients.
Chen 2021	RCT DB crossover	Trazodone	ArTh	1 night	22	Trazodone significantly increased the percentage SWS and improved AHI.
Smales 2015	RCT DB crossover	Trazodone	ArTh	1 night	15	Trazodone resulted in a significant reduction in AHI.
Messineo2020	RCT DB crossover	Zolpidem	ArTh	1 night	19	Zolpidem did not change AHI.
Carter 2016	RCT DB crossover	Zopiclone	ArTh	1 night	12	Zopiclone increased ArTh with no impact on AHI.
Carter 2018	RCT DB parallel	Zopiclone	ArTh	1 month	30	There was a tendency of reduction of AHI with zopiclone.
Carter 2020	RCT DB crossover	Zopiclone	ArTh	1 night	28	Zopiclone did not systematically reduce AHI or increased ArTh.

RCT, Randomized Clinical Trial, DB, Double-Blind; OL, Open-Label; MR, Muscle Responsiveness; LG, Loop Gain; ArTh, Arousal Threshold; UAA, Upper Airway Anatomy; AHI, Apnea-Hypopnea Index; AtoOxy, Atomoxetine + Oxybutynin; ReboxOxy, Reboxetine + Oxybutynin, OSA, Obstructive Sleep Apnea; HB, Hypoxic Burden; SWS, Slow Wave Sleep.

### *Upper airway anatomy*

A few different drug mechanisms can potentially target the collapsibility of the upper airway, such as weight loss medication that can reduce fat tissue on the tongue base and neck, diuretics reducing fluid retention, and nasal obstruction can be approached with intranasal steroids and decongestants.^6^

**Fig. 2:**
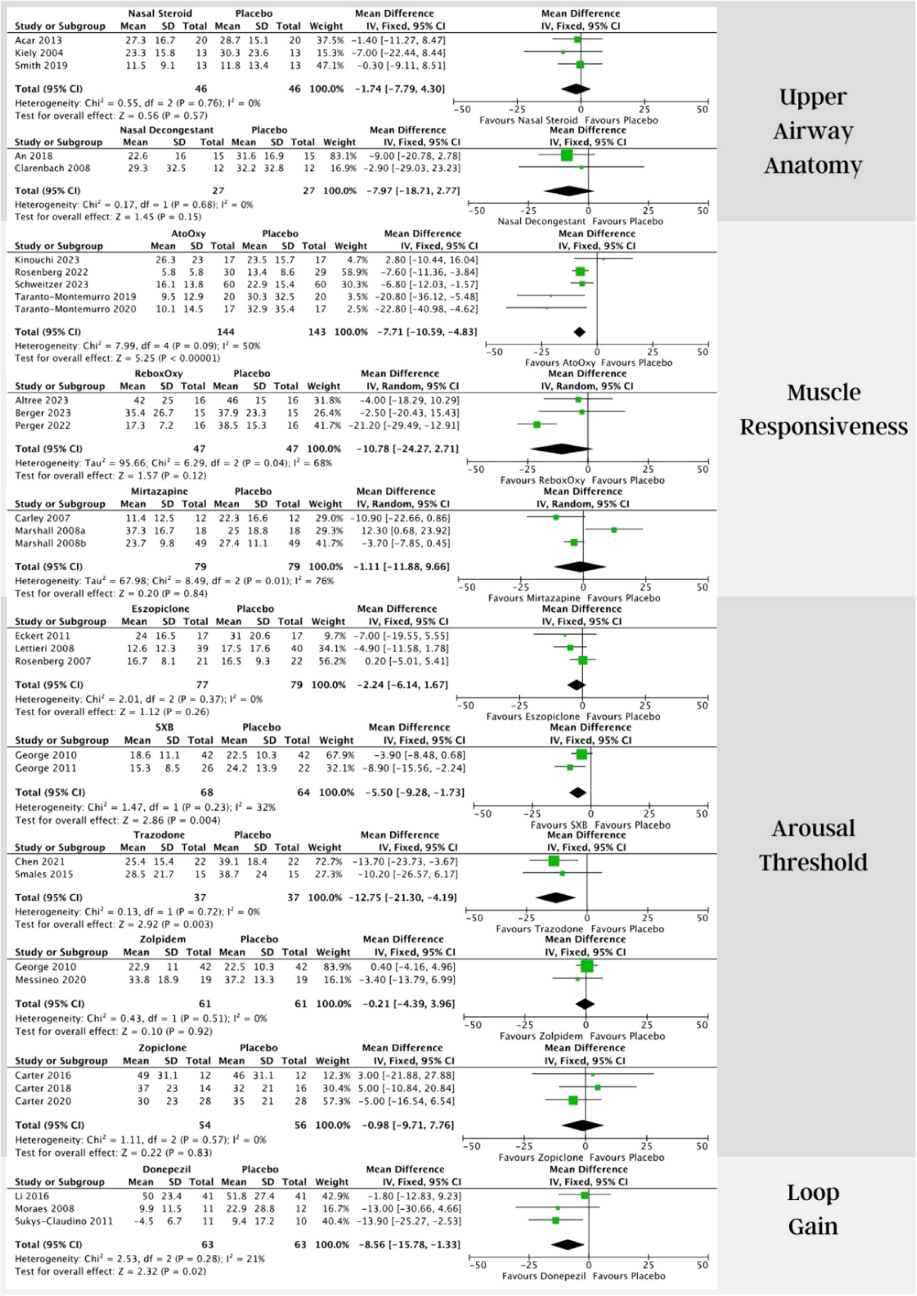
Forest plots illustrating apnea-hypopnea index mean difference in different drug therapies for obstructive sleep apnea against placebo.

Both the use of nasal steroids (3 studies) and, nasal decongestants (2 studies) versus placebo showed a tendency for improvement in AHI, without statistical significance (Fig. 2).^12^, ^13^, ^14^, ^15^, ^16^

### *Muscle responsiveness*

The combination of noradrenergic and antimuscarinic drugs was tested in different trials, Atomoxetine plus Oxybutynin (AtoOxy) showed significant improvement in AHI with combined data from 5 studies, mean difference of −7.71 (−10.59, −4.83) [Fixed, 95 % CI, I2 = 50 %, overall effect: *Z* = 5.25, *p* < 0.001].^17^, ^18^, ^19^, ^20^, ^21^ Reboxetine plus Oxybutynin (ReboxOxy) was assessed in 3 studies, and although there was a tendency for improvement, no significance was found (Fig. 2).^22^, ^23^, ^24^

Mirtazapine was tested by two authors in 3 trials, none of which evidenced the benefits of this drug treatment for OSA, moreover, one of these trials was discontinued due to trial failure and safety concerns.^25^^,^^26^

### *Arousal threshold*

Eszopiclone, zolpidem, and zopiclone were studied and showed no difference in AHI from placebo.^27^, ^28^, ^29^, ^30^, ^31^, ^32^, ^33^ Sodium Oxybate (SXB) and trazodone showed significant improvement in AHI. SXB vs placebo in AHI (2 studies, 90 patients) mean difference of −5.50 (−9.28, −1.73) [Fixed, 95 % CI, I2 = 32 %, overall effect: *Z* = 2.86, *p* = 0.004].^34^^,^^35^ Trazodone vs placebo in AHI (2 studies, 37 patients) mean difference of −12.75 (−21.30, −4.19) [Fixed, 95 % CI, I2 = 0 %, overall effect: *Z* = 2.92, *p* = 0.003].^36^^,^^37^

### *Loop gain*

Concerning loop gain, the only drug with enough studies that met inclusion criteria and could be combined into a meta-analysis was donepezil. Three studies assessed its effect on AHI against placebo demonstrating improvement in OSA severity, with a mean difference of −8.56 (−15.78, −1.33) [Fixed, 95 % CI, I2 = 21 %, overall effect: *Z* = 2.32, *p* = 0.02].^38^, ^39^, ^40^

### *Risk of bias assessment*

**Fig. 3:**
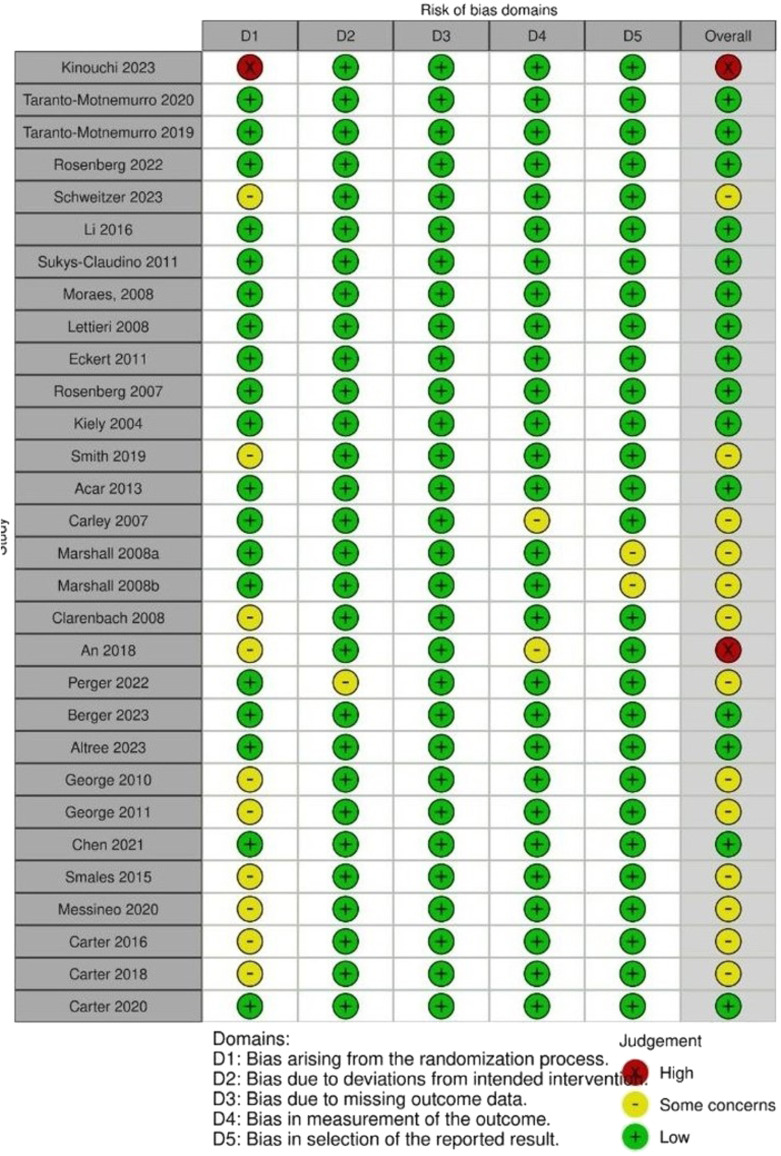
Risk of bias of the studies included in the meta-analysis.

**Fig. 4:**
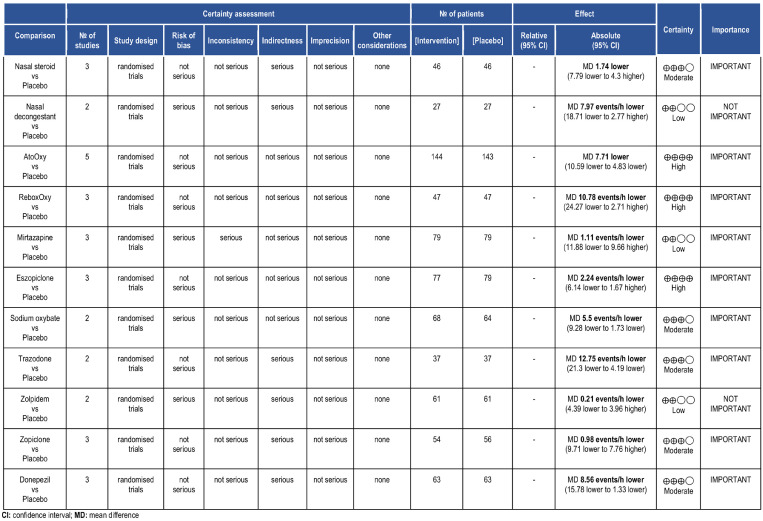
GRADE strength of the evidence assessment.

The majority of the studies included were double-blind randomized control trials with an overall low risk or with some concerns of bias (Fig. 3). The strength of the evidence was assessed by GRADE (Fig. 4).

## Discussion

The combination of atomoxetine and oxybutynin was found to provide the most significant enhancement in OSA severity.^17^, ^18^, ^19^, ^20^, ^21^ Nevertheless, all studies with this treatment were single-night studies with small sample sizes.

Historically, the use of drugs that would increase the arousal threshold in patients was thought to worsen apnea by decreasing muscle dilator response and promoting collapsibility. However, the use of zolpidem, eszopiclone, and zopiclone was found not to impact OSA severity compared to placebo.^27^, ^28^, ^29^, ^30^, ^31^, ^32^, ^33^ Moreover, sodium oxybate and trazodone showed improvement in AHI.^34^, ^35^, ^36^, ^37^

It is important to frame that this study only brings data from primary studies that met the defined inclusion criteria and could be combined in a meta-analysis. A limitation is that drugs that have been tested by a single RCT have not been included. There is also heterogeneity among populations included in different trials that were combined, such as different degrees of OSA severity which may impact drug efficacy.

Moreover, to better understand physio-pathological endotypes other outcomes such as loop gain, arousal threshold, muscle compensation, and hypoxic burden could be assessed.

## Conclusion

While numerous drugs have been investigated, only a few have shown promising results, like the combination of noradrenergic and antimuscarinic drugs. Identifying endotypes that respond to each pharmacological mechanism may be the key to future drug therapies for OSA. Moreover, studies with longer follow-up periods assessing the safety and sustained effects of these treatments are needed.

## Authors’ contributions

Nobre ML was responsible for the study conception and design, acquisition of data, analysis and interpretation of data, drafting of manuscript, and critical revision. Sarmento ACA, Oliveira PF, and Wanderley FF were responsible for the interpretation of data, drafting of manuscript, and critical revision. Diniz Júnior J was responsible for the study conception and design, drafting of the manuscript and critical revision. Gonçalves AK was responsible for the study conception and design, analysis, and interpretation of data, drafting of the manuscript and critical revision.

## Declaration of competing interest

The authors declare no conflicts of interest.

## References

[1] Chang J.L., Goldberg A.N., Alt J.A., Mohammed A., Ashbrook L., Auckley D. (2023). International consensus statement on obstructive sleep apnea. Int Forum Allergy Rhinol.

[2] Benjafield A.V., Ayas N.T., Eastwood P.R., Heinzer R., Ip M.S.M., Morrell M.J. (2019). Estimation of the global prevalence and burden of obstructive sleep apnoea: a literature-based analysis. Lancet Respir Med.

[3] Lloyd-Jones D.M., Allen N.B., Anderson C.A.M., Black T., Brewer L.P.C., Foraker R.E. (2022). American Heart Association. Life's essential 8: updating and enhancing the American Heart Association's construct of cardiovascular health: a presidential advisory from the American Heart Association. Circulation..

[4] Gottlieb D.J., Punjabi N.M. (2020). Diagnosis and management of obstructive sleep apnea: a review. JAMA.

[5] Kennedy B., Lasserson T.J., Wozniak D.R., Smith I. (2019). Pressure modification or humidification for improving usage of continuous positive airway pressure machines in adults with obstructive sleep apnoea. Cochrane Database Syst Rev.

[6] Taranto-Montemurro L., Messineo L., Wellman A. (2019). Targeting endotypic traits with medications for the pharmacological treatment of obstructive sleep apnea. A review of the current literature. J Clin Med.

[7] Eckert D.J., White D.P., Jordan A.S., Malhotra A., Wellman A. (2013 Oct 15). Defining phenotypic causes of obstructive sleep apnea. Identification of novel therapeutic targets. Am J Respir Crit Care Med.

[8] Mason M., Welsh E.J., Smith I. (2013). Drug therapy for obstructive sleep apnoea in adults. Cochrane Database Syst Rev.

[9] Page M.J., McKenzie J.E., Bossuyt P.M., Boutron I., Hoffmann T.C., Mulrow C.D. (2021). The PRISMA 2020 statement: an updated guideline for reporting systematic reviews. BMJ.

[10] Sterne J.A.C., Savović J., Page M.J., Elbers R.G., Blencowe N.S., Boutron I. (2019). RoB 2: a revised tool for assessing risk of bias in randomised trials. BMJ.

[11] Guyatt G., Oxman A.D., Akl E.A., Kunz R., Vist G., Brozek J. (2011). GRADE guidelines. GRADE guidelines: 1. Introduction-GRADE evidence profiles and summary of findings tables. J Clin Epidemiol.

[12] Kiely J.L., Nolan P., McNicholas W.T. (2004). Intranasal corticosteroid therapy for obstructive sleep apnoea in patients with co-existing rhinitis. Thorax.

[13] Smith D.F., Sarber K.M., Spiceland C.P., Ishman S.L., Augelli D.M., Romaker A.M. (2019). Effects of medical therapy on mild obstructive sleep apnea in adult patients. J Clin Sleep Med.

[14] Acar M., Cingi C., Sakallioglu O., San T., Yimenicioglu M.F., Bal C. (2013). The effects of mometasone furoate and desloratadine in obstructive sleep apnea syndrome patients with allergic rhinitis. Am J Rhinol Allergy.

[15] Clarenbach C.F., Kohler M., Senn O., Thurnheer R., Bloch K.E. (2008). Does nasal decongestion improve obstructive sleep apnea?. J Sleep Res.

[16] An Y., Li Y., Kang D., Sharama-Adhikari S.K., Xu W., Li Y (2019). The effects of nasal decongestion on obstructive sleep apnoea. Am J Otolaryngol.

[17] Kinouchi T., Terada J., Sakao S., Koshikawa K., Sasaki T., Sugiyama A. (2023). Effects of the combination of atomoxetine and oxybutynin in Japanese patients with obstructive sleep apnoea: a randomized controlled crossover trial. Respirology.

[18] Taranto-Montemurro L., Messineo L., Azarbarzin A., Vena D., Hess L.B., Calianese N.A. (2020). Effects of the combination of atomoxetine and oxybutynin on OSA endotypic traits. Chest.

[19] Taranto-Montemurro L., Messineo L., Sands S.A., Azarbarzin A., Marques M., Edwards B.A. (2019). The combination of atomoxetine and oxybutynin greatly reduces obstructive sleep apnea severity. a randomized, placebo-controlled, double-blind crossover trial. Am J Respir Crit Care Med.

[20] Rosenberg R., Abaluck B., Thein S. (2022). Combination of atomoxetine with the novel antimuscarinic aroxybutynin improves mild to moderate OSA. J Clin Sleep Med.

[21] Schweitzer P.K., Maynard J.P., Wylie P.E., Emsellem H.A., Sands S.A. (2023). Efficacy of atomoxetine plus oxybutynin in the treatment of obstructive sleep apnea with moderate pharyngeal collapsibility. Sleep Breath.

[22] Perger E., Taranto Montemurro L., Rosa D., Vicini S., Marconi M., Zanotti L. (2022). Reboxetine Plus Oxybutynin for OSA Treatment: a 1-Week, Randomized, Placebo-Controlled, Double-Blind Crossover Trial. Chest.

[23] Berger M., Solelhac G., Marchi N.A., Dussez R., Bradley B., Lecciso G. (2023). Effect of oxybutynin and reboxetine on obstructive sleep apnea: a randomized, placebo-controlled, double-blind, crossover trial. Sleep.

[24] Altree T.J., Aishah A., Loffler K.A., Grunstein R.R., Eckert D.J. (2023). The norepinephrine reuptake inhibitor reboxetine alone reduces obstructive sleep apnea severity: a double-blind, placebo-controlled, randomized crossover trial. J Clin Sleep Med.

[25] Carley D.W., Olopade C., Ruigt G.S., Radulovacki M. (2007). Efficacy of mirtazapine in obstructive sleep apnea syndrome. Sleep.

[26] Marshall N.S., Yee B.J., Desai A.V., Buchanan P.R., Wong K.K.H., Crompton R. (2008). Two randomized placebo-controlled trials to evaluate the efficacy and tolerability of mirtazapine for the treatment of obstructive sleep apnea. Sleep.

[27] Lettieri C.J., Quast T.N., Eliasson A.H., Andrada T. (2008). Eszopiclone improves overnight polysomnography and continuous positive airway pressure titration: a prospective, randomized, placebo-controlled trial. Sleep.

[28] Eckert D.J., Owens R.L., Kehlmann G.B., Wellman A., Rahangdale S., Yim-Yeh S. (2011). Eszopiclone increases the respiratory arousal threshold and lowers the apnoea/hypopnoea index in obstructive sleep apnoea patients with a low arousal threshold. Clin Sci (Lond).

[29] Rosenberg R., Roach J.M., Scharf M., Amato D.A. (2007). A pilot study evaluating acute use of eszopiclone in patients with mild to moderate obstructive sleep apnea syndrome. Sleep Med.

[30] Messineo L., Eckert D.J., Lim R., Chiang A., Azarbarzin A., Carter S.G. (2020). Zolpidem increases sleep efficiency and the respiratory arousal threshold without changing sleep apnoea severity and pharyngeal muscle activity. J Physiol.

[31] Carter S.G., Berger M.S., Carberry J.C., Bilston L.E., Butler J.E., Tong B.K.Y. (2016). Zopiclone increases the arousal threshold without impairing genioglossus activity in obstructive sleep apnea. Sleep..

[32] Carter S.G., Carberry J.C., Cho G., Fisher L.P., Rollo C.M., Stevens D.J. (2018). Effect of 1 month of zopiclone on obstructive sleep apnoea severity and symptoms: a randomised controlled trial. Eur Respir J.

[33] Carter S.G., Carberry J.C., Grunstein R.R., Eckert D.J. (2020). Randomized trial on the effects of high-dose zopiclone on OSA severity, upper airway physiology, and alertness. Chest.

[34] George C.F., Feldman N., Inhaber N., Steininger T.L., Grzeschik S.M., Lai C. (2010). A safety trial of sodium oxybate in patients with obstructive sleep apnea: acute effects on sleep-disordered breathing. Sleep Med.

[35] George C.F., Feldman N., Zheng Y., Steininger T.L., Grzeschik S.M., Lai C. (2011). A 2-week, polysomnographic, safety study of sodium oxybate in obstructive sleep apnea syndrome. Sleep Breath.

[36] Chen C.Y., Chen C.L., Yu C.C. (2021). Trazodone improves obstructive sleep apnea after ischemic stroke: a randomized, double-blind, placebo-controlled, crossover pilot study. J Neurol.

[37] Smales E.T., Edwards B.A., Deyoung P.N., McSharry D.G., Wellman A., Velasquez A. (2015). Trazodone effects on obstructive sleep apnea and non-REM arousal threshold. Ann Am Thorac Soc.

[38] Li Y., Owens R.L., Sands S., Orr J., Moraes W., DeYoung P. (2016). The effect of donepezil on arousal threshold and apnea-hypopnea index. A randomized, double-blind, cross-over study. Ann Am Thorac Soc.

[39] Sukys-Claudino L., Moraes W., Guilleminault C., Tufik S., Poyares D. (2012). Beneficial effect of donepezil on obstructive sleep apnea: a double-blind, placebo-controlled clinical trial. Sleep Med.

[40] Moraes W., Poyares D., Sukys-Claudino L., Guilleminault C., Tufik S. (2008). Donepezil improves obstructive sleep apnea in Alzheimer disease: a double-blind, placebo-controlled study. Chest.

